# Relationship Between Patient State Index and Richmond Agitation Sedation Scale for Sedation in Critically Ill Patients: An Observational Analytical Study

**DOI:** 10.4274/TJAR.2026.262486

**Published:** 2026-06-26

**Authors:** Nishant Kumar, Kritika Tiwari, Maitree Pandey

**Affiliations:** 1 Lady Hardinge Medical College and Associated Hospitals Department of Anaesthesiology and Critical Care, New Delhi, India

**Keywords:** Deep sedation, fentanyl, intensive care unit, midazolam, morphine, receiver operating curve

## Abstract

**Objective:**

The objective of this study was to find the relationship between Patient State Index (PSI) and Richmond Agitation Sedation Scale (RASS) for sedation in critically ill patients.

**Methods:**

This was a prospective, observational study. Thirty-five patients were recruited to assess the correlation between PSI and RASS scores of 0 to -3 for sedation in mechanically ventilated, critically ill patients. Paired observations (RASS and PSI) were made for each patient every 4 hours for at least 72 hours or until discontinuation of monitoring, whichever occurred earlier. Appropriate statistical analyses were applied; a *P* < 0.05 was considered significant.

**Results:**

Out of the expected 665 pairs of observations, only 608 pairs were observed. The median PSI value with all sedation regimen was 72 with an interquartile range of 60 and 86 (1^st^ and 3^rd^ quartile) respectively. There was significant and strong correlation between PSI and RASS 0 to -3 with Spearman correlation coefficient of 0.822; R^2^=0.675 (*P* < 0.001) which dropped to 0.786 with repeated measures correlation. The sensitivity and specificity were 88.66% and 88.57%, respectively, with an area under the receiver operating characteristic curve of 0.947; these improved to 100% and an area under the curve of 1 when analysed per patient. To maintain RASS between 0 and -3, the PSI cutoff was found to be 50-52.

**Conclusion:**

PSI correlates well with RASS across sedation regimens in critically ill patients and assists in monitoring of sedation. Adequate sedation to reach an RASS of 0 to -3 may be achieved at a PSI of 50-52. However, these findings are preliminary and require validation in larger cohorts.

**Main Points**

• Patient State Index (PSI) can be used to monitor sedation continuously in the intensive care unit.

• There is a strong and statistically significant correlation between PSI and Richmond Agitation Sedation Scale (RASS) scores from 0 to -3, with a Spearman correlation coefficient of 0.822.

• PSI correlates well with RASS across all sedation regimens in critically ill patients.

• Adequate sedation to achieve a RASS of 0 to -3 may be achieved at a PSI 50-52.

## Introduction

Critically ill patients or those undergoing major surgery frequently require mechanical ventilation as part of their care. Appropriate administration of analgesia and sedation is important for patient comfort, safety, and synchrony with mechanical ventilation. The level of sedation should be titrated to each individual’s requirements to allow a shorter duration of mechanical ventilation, a shorter hospital stay, and reduced mortality.^1^ Off-target sedation results in excessive pain, anxiety, and agitation; self-removal of tubes and catheters; violence toward caregivers; myocardial ischemia; and hypoxemia. In contrast, excessive or prolonged sedation can lead to skin breakdown, nerve compression, delirium, long-term cognitive dysfunction, ventilator-associated pneumonia, posttraumatic stress disorder, and other complications. Different scoring systems are used for the assessment of sedation, such as Sedation Agitation Score (SAS), Glasgow Coma Scale (GCS), Ramsay Sedation Score (RSS), and Richmond Agitation Sedation Scale (RASS). Sedation scales are used to assess depth of sedation, wakefulness, arousal in response to stimuli, and cognition.^2^

RASS is the most commonly used tool for assessing level of sedation in intensive care unit (ICU) patients. It is a subjective 10-point scale with four levels indicating anxiety or agitation, one level denoting a calm and alert state, and 5 levels indicating sedation (-5 to +5); it has been validated for interrater reliability in the ICU. A RASS score between -3 and 0 should be maintained in the ICU.^3^ However, as with others, it depends on the user’s interpretation and is not continuous. Electroencephalogram (EEG)-based depth of anaesthesia monitors, such as bispectral index (BIS), Narcotrend, CONOX, Entropy, and PSI, on the other hand, provide a continuous measurement of the depth of sedation.

The PSI is a dimensionless number ranging from 100 (fully awake) to 0 (deeply anaesthetised), based on the analysis of four-channel EEG, and inversely reflects the level of sedation and hypnosis. PSI has been used extensively in operating theatres to measure depth of anaesthesia and can be utilised in the ICU to measure the level of sedation continuously without applying arousal stimuli, and hence may allow the maintenance of a more constant level of sedation with continuous rather than intermittent monitoring.^4^ The PSI is used to assess sedation, but there is no defined PSI value for sedation in critically ill patients. Currently, few studies have examined the use of PSI to evaluate depth of sedation in the ICU and to compare its performance with established sedation scores. Although PSI values during anaesthesia have been defined as between 25 and 50 to ensure adequate depth, no such values have been defined for ICU sedation. The range of PSI corresponding to RASS scores of 0 to -3 remains unknown. PSI may better reflect depth of sedation, as it aggregates 4 waveforms (2 each bilaterally) and thereby reflects both the dominant and non-dominant hemispheres compared with the BIS or entropy, which are unilateral and should ideally be applied contralaterally to the dominant hand. Therefore, this study was planned to investigate the relationship between PSI and RASS and to determine the PSI corresponding to RASS 0 to -3 in critically ill patients.

## Methods

This prospective observational study was approved by the Institutional Ethics Committee of Lady Hardinge Medical College and Affiliated Hospitals, Shahid Bhagat Singh Marg, New Delhi, India (approval no.: LHMC/IEC/2023/PG Thesis/4/R, date: 06.05.2023). The study was pre-registered in the Indian Clinical Trials Registry (CTRI registration number CTRI/2023/06/053988) on June 16, 2023. All procedures were carried out in accordance with the standards of the institutional ethical committee on human experimentation and with the Declaration of Helsinki (1975) and its 2013 revision. Patients of either sex aged more than 18 years who were admitted to the ICU were included in the study. Patients receiving muscle relaxants, those with head injury, focal brain disease, or abnormal electrical brain activity were excluded from the study. A written, informed, voluntary consent for participation in the study was obtained from the patient, next of kin, or authorized signatory after carefully explaining the procedure and the need for the study in their own language. A careful and detailed history and physical examination were performed, including the patient’s current treatment and medication history. Patients were sedated according to the regimen advised by the treating physician. The drugs used for sedation were recorded.

After skin preparation with alcohol, PSI electrodes were attached to specified locations on the forehead. Adequate electrode contact, as depicted on the monitor, was maintained throughout the study. The PSI value was recorded on a Root model Masimo SET monitor every 4 hours after confirming adequate contact, signal quality, and artifact <10%. The value of PSI was recorded either before any stimulus to the patient or after the stimulus-at least 15 minutes later or when the PSI value had stabilized; stimuli included tube suction, change of dressing or bed linen, or a recent bolus of sedative. The trained attending ICU resident on duty recorded the PSI values before noting the RASS. RASS was observed and rated as described in Table 1. Both readings were noted every 4 hours for at least 72 hours or until discontinuation of monitoring, whichever occurred earlier.

Based on a study by Abouelela and Abdelazim,^1^ with a correlation coefficient of 0.686 between BIS and RASS in ICU patients, with an α=0.05 and β=0.01, sample size was calculated to be 29 patients. Assuming a 20% dropout rate, 35 patients were recruited for the study.

### Statistical Analysis

The data obtained were entered into a Microsoft Excel worksheet and analysed using IBM Statistical Package for Social Sciences (SPSS), USA, version 29. The Spearman correlation coefficient was calculated to assess the relationship between PSI and RASS. As a secondary analysis to account for repeated measures within patients, a repeated-measures Spearman correlation was performed using Jamovi version 2.7.17 (retrieved from https://www.jamovi.org). Correlation values between 0 and 0.20 were considered negligible; values between 0.21 and 0.40 were considered weak; values between 0.41 and 0.60 were considered moderate; values between 0.61 and 0.80 were considered strong; and values between 0.81 and 1 were considered very strong. The value of PSI corresponding to RASS 0 to -3 was obtained using receiver operating characteristic (ROC) curve analysis. Further sub-analysis was performed by categorising the data based on the sedation regimen used. Individual relationships between PSI and RASS and cut-offs of PSI with respect to the sedation regimen were also calculated. To account for repeated measures within patients, the PSI was averaged for RASS 0 to -3 and for values beyond this range. ROC was recalculated using the patient as a unit rather than the total number of observations.

## Results

**Figure 1:**
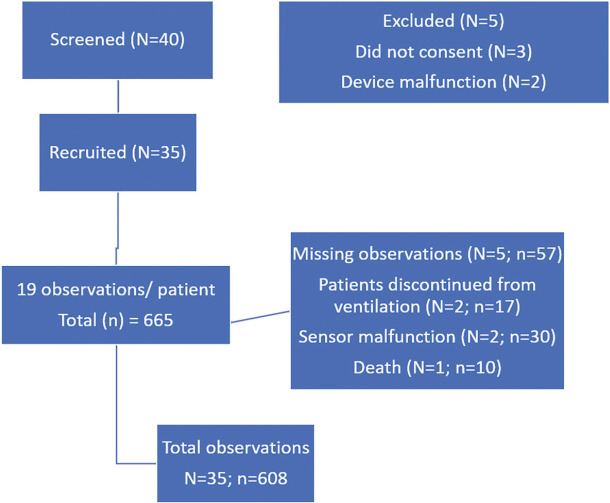
Flow of patients.

Forty patients were screened for the study; five were excluded: the relatives of three patients did not consent to the study, and for two patients the device was not functioning due to a technical problem. A total of 35 patients were recruited. Based on the observation interval, 19 paired observations were to be made for each patient. Out of the expected 665 pairs of observations, only 608 pairs (n = 608) were observed because two patients were discontinued from mechanical ventilation within 72 hours; for these patients only 10 and 11 observations, respectively, could be recorded. In two other patients, only 4 observations per patient could be noted due to sensor malfunction. In one patient, only 9 observations could be noted, as the patient expired before the completion of our study (Figure 1). The patient characteristics are described in Table 2.

Of the 608 observed pairs, only 538 were within RASS 0 to -3, indicating that the patients were oversedated at 70 time points (Table 3).

The Spearman correlation (ρ) between PSI and RASS with all sedation regimen was found to be 0.822 with an R^2^ of 0.675 (Table 4).

**Figure 2:**
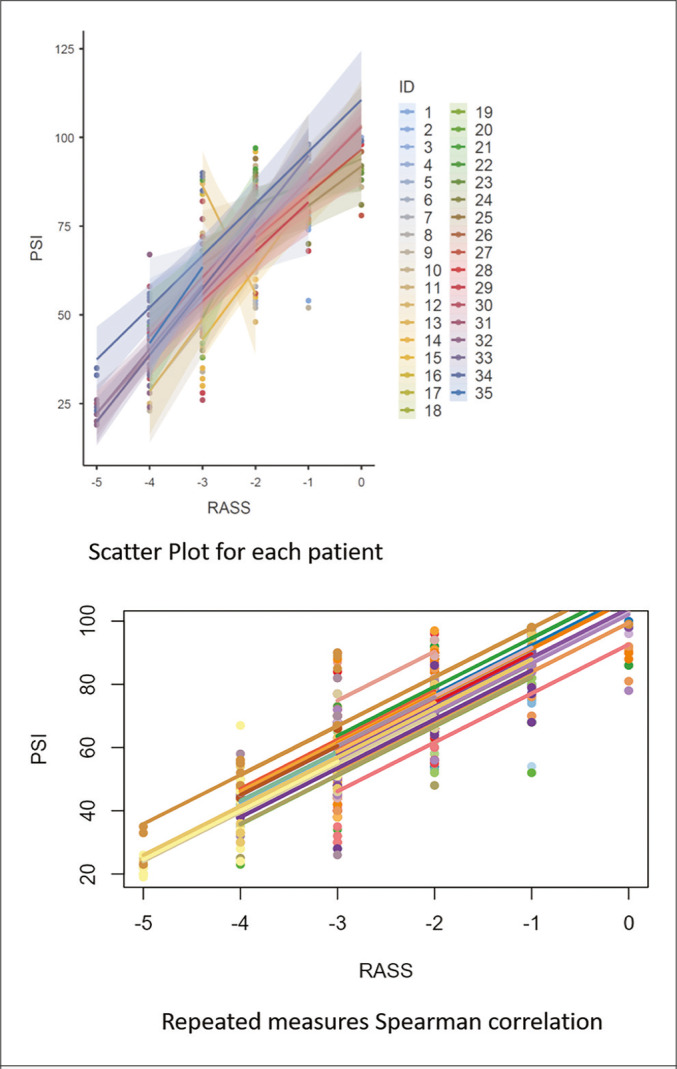
Repeated measures correlation. PSI, Patient State Index; RASS, Richmond Agitation Sedation Scale.

To account for within-patient clustering, a repeated-measures Spearman correlation was performed with each patient treated as a cluster. The Spearman correlation (ρ) between PSI and RASS was 0.786 (95% CI: 0.753, 0.816; *P* < 0.001), indicating a strong correlation (Figure 2).

ROC analyses were carried out to identify the cutoff PSI value corresponding to RASS scores 0 to -3, with an AUC of 0.94 sq units (Table 5). The cutoff PSI value was 50.5 for all sedation regimens, suggesting that at a PSI of 50.5, the RASS would be between 0 and -3, and the patient would be adequately sedated with any of the regimens. Similar ROC curves were also plotted to determine PSI cutoffs for predicting RASS between 0 and -3 for each sedation regimen (Table 5). However, accounting for repeated measures for the same patient, PSI was averaged for RASS groups and ROC reperformed. A PSI of 51.4 corresponds to RASS scores 0 to -3, with an AUC of 1. Table 5 describes ROC characteristics for data points treated as independent observations (n) and for averaged data per patient (N).

## Discussion

This prospective observational study (n = 608) aimed to determine the relationship between PSI and RASS during sedation in critically ill patients. Our study suggests a very strong correlation (ρ-0.822, R^2^=0.67; *P *< 0.001) between PSI and RASS. Of the 608 observations, patients were oversedated at 70 time points (RASS<-3). A PSI >50.5 corresponds to RASS scores of 0 to -3, with both sensitivity and specificity of 88.6%. However, in repeated-measures analysis, the correlation remained strong (ρ-0.786; *P* < 0.001). The corresponding PSI for RASS scores of 0 to -3 was 51.4, with both sensitivity and specificity at 100%. Since the sample size for subgroup analyses was small, the root-mean-square differences between the estimated and true metrics may be considerable, with weak correlation and poor regression fit between the estimated and true metrics.

Sedation during mechanical ventilation is important not only to relieve stress, anxiety, pain, and suffering, but also to facilitate patient- ventilator interaction and synchrony and to minimize potential adverse effects related to mechanical ventilation. Excessive or prolonged sedation can lead to skin breakdown, nerve compression, delirium, long-term cognitive dysfunction, ventilator-associated pneumonia, post-traumatic stress disorder, and other complications. Inadequate sedation may result in excessive pain, anxiety, agitation, self-removal of tubes and catheters, violence towards caregivers, myocardial ischemia, and hypoxemia.^2^

Conventionally, sedation has been assessed using clinical signs, such as patient response to a stimulus (which may be masked if paralysis has been achieved), hemodynamic parameters (heart rate, non-invasive blood pressure), and subjective scales such as the RSS, Sedation Agitation Scoring System, RASS. A RASS score of 0 to -3 is considered adequate for sedation and to prevent recall. However, they are not continuous, and inter-user variability exists. A RASS value between -3 and 0 indicates light to moderate sedation; -4 and -5 indicate oversedation, while a RASS value >0 indicates insufficient sedation. RASS is the first sedation scale validated for its ability to detect changes in sedation status in ICU patients.^3^

Since most sedative agents elicit a neurohormonal response in the cerebral cortex, changes from wakefulness to sleep and unconsciousness can be monitored using EEG. During relaxation with the eyes closed, a predominance of alpha waves (7.5-12.50 Hz) is observed. Light sedation causes a decrease in alpha power and an increase in beta power (12.530 Hz). With deepening of sedation, slow-wave activity—specifically delta (1.5-3.5 Hz) and theta (3.5-7.5 Hz) waves—increases and becomes more prominent, accompanied by a spontaneous decrease in alpha and beta activity in all regions. This represents a decrease in cortical generators of alpha and beta activity, with a shift towards control by thalamo-hippocampal-septal generators of delta and theta activity.^5^ The EEG requires placement of 32 electrodes across the skull, but its interpretation is difficult, and continuous monitoring is not possible.

To simplify the interpretation of EEG waves, processed EEG was developed to study the effects of various drugs on the brain. The transition from awake to unconscious state is accompanied by changes in the brain’s spontaneous electrical activity, which can be recorded by electrodes placed on the forehead, analyzed, and converted into a number. This has led to the development of monitors, such as PSI, to measure the depth of sedation and anaesthesia. PSI is a composite EEG measure that correlates with behavioral assessments of sedation and hypnosis. The PSI was developed using an algorithm; values range from 0 to 100, with 25-50 signifying surgical anaesthesia and a low incidence of recall.^4^ However, no such value has been defined for assessing sedation in critically ill patients.

All the drugs used for sedation in the ICU affect cognitive function and memory, with resultant characteristic changes on EEG. Benzodiazepines such as midazolam bind to gamma-aminobutyric acid (GABA) A receptors and enhance the ability of endogenous GABA to open the channel by inducing a rotational conformational change that increases chloride conductance through the receptor. This results in neuronal hyperpolarization and reduced neuronal excitability.^6^ The most important effects are sedative-hypnotic and amnestic properties. Midazolam increases delta and beta power, whereas alpha power is significantly decreased. However, midazolam decreases cerebral blood flow, and the resultant changes are reflected in a shift of EEG power toward lower frequencies. It acts on the GABA-A receptor to reduce the excitability of neurons, resulting in impairment of multidomain cognitive functions: it impairs the encoding of new information, has no deleterious effect on either retention or retrieval of information acquired before drug administration, and leads to a dose-dependent decrease in PSI.^7^

Opioids, such as morphine and fentanyl, on the other hand, produce their main pharmacologic effects by interacting with opioid receptors, which are G protein-coupled. Binding of opioid agonists to these receptors leads to activation of the G protein, producing primarily inhibitory effects at the neuronal level. These effects ultimately lead to reduced presynaptic neurotransmi tter release and postsynaptic hyperpolarization of neurons.^8^ The relief of pain is the primary therapeutic effect of opioids. Opioids mediate their effects via opioid receptors: mu, delta, and kappa. They act on spinal and brain mu receptors and provide analgesia by attenuating the nociceptive fibres and altering the affective response to painful stimulation. However, as the dose increases, mu agonism produces drowsiness and sleep by inducing changes in subcortical brain areas. They produce delta wave activity that, on the EEG, resembles the pattern observed during natural sleep. Opioids at their analgesic doses produce minimal electrophysiological changes in the cerebral cortex and subcortical areas, whereas higher concentrations of opioids are required to induce EEG changes. They produce significant pain relief at doses that do not induce sleep.^9^

Fentanyl induces frontal theta-wave activity, which leads to deep relaxation and consequently to the lowest PSI values, since the major EEG signal is obtained from the frontal cortex. A small dose of fentanyl (2-5 µg kg^-1^) produces minimal EEG changes, whereas a high dose (30-70 µg kg^-1^) produces high-voltage slow waves (δ), resulting in sedation.^8^ Although we used a low dose of fentanyl (2 µg kg^-1^), the resultant median PSI was the highest, while the cutoff (41.5) corresponding to RASS 0 to -3 was the lowest among the sedation regimens used, indicating increased suppression of the EEG, which is contrary to known theories. This may be due to patients included in this regimen being sicker; a larger heterogeneous sample may be required to prove or disprove these findings.

Morphine reduces high frequency β_1 _and β_2 _EEG powers and decrease coherence between frontal and occipital activity indicating that morphine induces a deep state of sedation, which is reflected by the lowest median values, strong correlation and association and a resultant PSI cutoff of >57, similar to that of midazolam.^8^ In the critically ill, active metabolite and decreased renal clearance can be additional factors leading to enhanced drug effects.

Deogaonkar et al.^10^ compared BIS with three commonly used SAS (RASS/SAS/GCS) in brain injury patients. The study showed a strong correlation between the BIS and RASS (R^2^=0.810; *P* < 0.0001), SAS score (R^2^=0.725; *P* < 0.0001), and GCS score (R^2^=0.655; *P* < 0.0001). This correlation was present regardless of whether the patients received any sedative medications. Thus, processed EEG may accurately mimic the established scales and provide continuous monitoring of underlying consciousness.

However, little difference was observed when midazolam and morphine were administered together. The cut-off value for PSI to maintain RASS 0 to -3 was >55 versus >50.5 for midazolam alone. This further augurs the fact that suppression of consciousness at analgesic doses is not a property that can be attributed to opioids.

Although studies have compared BIS and entropy with other sedation scales, we found no literature regarding the use of PSI and RASS for sedation in ICU patients. The relationship between continuous monitors and objective scales has been observed to be poor or, at best, moderate.

When SAS was compared with BIS in critically ill patients receiving lorazepam infusions, the two showed a poor correlation (r^2^=0.006). Moreover, the plasma concentrations of lorazepam also did not correlate with BIS (r^2^=0.30).^11^ Similarly, state entropy showed a poor correlation (r=0.33) with RASS when propofol and fentanyl were used for sedation in critically ill patients.^12^

However, Arbour et al.^13^ found a moderately positive correlation between BIS and SAS (r=0.50 and R^2^=0.252) suggesting that the BIS explained only 25.2% of the variance in SAS score. This suggests that, when clinical assessment is equivocal, BIS may have an adjunctive role in assessing sedation.

Contrary to the above the studies, our results were similar to that by Abouelela and Abdelazim,^1^ who reported a consistent and strong correlation of RASS with BIS (r=0.611-0.699) using midazolam for sedation and fentanyl infusion for analgesia in the critically ill. This was not replicated using dexmedetomidine as a sedative (r=0.011 to 0.514).

We found a very strong correlation between RASS scores from 0 to -3 and PSI, indicating that the two measures mirror each other. PSI has the added advantage of providing continuous monitoring, which helps prevent oversedation, detect acute changes in consciousness, and identify EEG alterations due to spike activity (e.g., absence or focal seizures) that may otherwise go unnoticed.

The strengths of our studies are that, at present few studies have compared PSI and sedation scales in ICU patients. Although other continuous monitors have been used, none specifies the processed EEG value that should be maintained for appropriate sedation in critically ill patients.

### Study Limitations

Our study is not without limitations. Although a strong correlation and association were observed between RASS and PSI, this could be confounded by patients’ underlying conditions and different sedation regimens. Small subgroup sizes limit the reliability of subgroup analyses and may produce unstable statistical estimates; hence, these analyses should be interpreted with caution. This was addressed by excluding patients with expected neurological deficits or muscle relaxation, and by performing a further sub-analysis of the sedation regimens, which yielded the same results.

## Conclusion

PSI correlates well with RASS across sedation regimens in critically ill patients and assist in monitoring of sedation. Adequate sedation to achieve a RASS of 0 to -3 may be achieved at a PSI 50-52. However, these findings are preliminary and require validation in larger cohorts.

## Ethics

**Ethics Committee Approval: **This prospective observational study was approved by the Institutional Ethics Committee of Lady Hardinge Medical College and Affiliated Hospitals, Shahid Bhagat Singh Marg, New Delhi, India (approval no.: LHMC/IEC/2023/PG Thesis/4/R, date: 06.05.2023).

**Informed Consent: **A written, informed, voluntary consent for participation in the study was obtained from the patient, next of kin, or authorized signatory after carefully explaining the procedure and the need for the study in their own language.

## Figures and Tables

**Table 1. Richmond Agitation-sedation Scale:** 

**Score**	**Term**	**Description**
**+4**	**Combative**	Overtly combative or violent; immediate danger to staff
**+3**	**Very agitated**	Pulls on or removes tube(s) or catheter(s) or has aggressive behavior toward staff
**+2**	**Agitated**	Frequent non-purposeful movement or patient-ventilator dyssynchrony
**+1**	**Restless**	Anxious or apprehensive but movements not aggressive or vigorous
**0**	**Alert and calm**	Spontaneously pays attention to caregiver
**-1**	**Drowsy**	Not fully alert, but has sustained (more than 10 seconds) awakening, with eye contact, to voice
**-2**	**Light sedation**	Briefly (less than 10 seconds) awakens with eye contact to voice
**-3**	**Moderate sedation**	Any movement (but no eye contact) to voice
**-4**	**Deep sedation**	No response to voice, but any movement to physical stimulation
**-5**	**Unarousable**	No response to voice or physical stimulation

**Table 2. Patient Characteristics:** 

**Age (years)**	**Median (IQR)**	46 (30, 60)
	**Mean** **±** **SD**	46.49±16.55
**Gender** **N (%)**	**Male**	13 (37%)
	**Female**	22 (63%)
**Daignosis** **N (%)**	**Liver abscess**	3 (12%)
	**Acute appendicitis**	1 (2.8%)
	**Buccal mucosal carcinoma**	1 (2.8%)
	**Colon cancer**	1 (2.8%)
	**Gall bladder cancer**	1 (2.8%)
	**Acute cholecystitis**	1 (2.8%)
	**Choledocholithiasis**	1 (2.8%)
	**Duodenal stricture**	1 (2.8%)
	**Morbid obesity**	2 (5.7%)
	**Multinodular goitre**	1 (2.8%)
	**Intestinal obstruction**	2 (5.7%)
	**Whipple’s procedure**	1 (2.8%)
	**Perforation peritonitis**	10 (28.5%)
	**Hemorrhagic changes and anemia in pregnancy**	9 (25.7%)
**Comorbidities** **N (%)**	**Hypertension**	9 (22%)
	**Diabetes mellitus**	7 (17%)
	**Hypothyroidism**	3 (7%)
	**Obesity**	1 (3%)
	**CVS/CLD/renal**	3 (2%)
	**No- comorbidities**	19 (45%)

IQR, interquartile range; SD, standard deviation; CVS, cardiovascular system; CLD, chronic liver disease

**Table 3. Distribution table: RASS 0 to -5:** 

**RASS**	**-5**	**-4**	**-3**	**-2**	**-1**	**0**
**n**	13	57	188	230	103	17
**Total**	**70 (oversedation)**		**538 (desired sedation)**			

RASS, Richmond Agitation Sedation Scale

**Table 4. Relationship Between PSI and RASS:** 

**Sedation regimen**	**Observations**						
**Total (all sedation)** **(N = 35/ n = 608)**	**Spearman’s rho**		**R^2^**	**Trend (X=RASS)**			***P* value**
	0.822		0.675	15.23x+103.38			**<0.001**
	**RASS**	**-5**	**-4**	**-3**	**-2**	**-1**	**0**
	**n**	13	57	188	230	103	17
	**Median**	22	41	56	75	90	92
	**Q1**	20	35	48	68	84	89
	**Q3**	26	48	66	84	94	98
**Midazolam** **(N = 16/n = 257)**	**Spearman’s rho**		**R^2^**	**Trend (X=RASS)**			***P* value**
	0.806		0.604	15.71x+105.71			**<0.001**
	**RASS**	**-5**	**-4**	**-3**	**-2**	**-1**	**0**
	**n**	0	8	84	114	42	9
	**Median**	-	43	56	76.5	90.5	96
	**Q1**	-	40	47	68	89	87
	**Q3**	-	53	66	87.25	95	98
**Midazolam + morphine** **(N = 6/n = 104)**	**Spearman’s rho**		**R^2^**	**Trend (X=RASS)**			***P* value**
	0.823		0.674	13.39x+100.77			**<0.001**
	**RASS**	**-5**	**-4**	**-3**	**-2**	**-1**	**0**
	**n**	3	23	28	37	9	4
	**Median**	33	45	62	75	88	90.5
	**Q1**	23	40	56	68	78	90
	**Q3**	-	50	70	80	94	97
**Midazolam + fentanyl** **(N = 2/n = 38)**	**Spearman’s rho**		**R^2^**	**Trend (X=RASS)**			***P* value**
	0.71		0.538	11.82x+92.897			**<0.001**
	**RASS**	**-5**	**-4**	**-3**	**-2**	**-1**	**0**
	**n**	0	0	16	15	5	2
	**Median**	-		53.5	68	88	86.5
	**Q1**	-		48.5	66	73	81
	**Q3**	-		67	75	89	**-**
**Morphine** **(N = 8/n = 152)**	**Spearman’s rho**		**R^2^**	**Trend (X=RASS)**			***P* value**
	0.872		0.768	16.55x+105.28			**<0.001**
	**RASS**	**-5**	**-4**	**-3**	**-2**	**-1**	**0**
	**n**	10	24	51	45	21	1
	**Median**	22	38	56	75	86	99
	**Q1**	20	33	48	66	80	99
	**Q3**	24.25	45.25	62	83	94	99
**Fentanyl** **(N = 3/n = 57)**	**Spearman’s rho**		**R^2^**	**Trend (X=RASS)**			***P* value**
	0.774		0.651	17.15x+102.70			**<0.001**
	**RASS**	**-5**	**-4**	**-3**	**-2**	**-1**	**0**
	**n**	0	2	9	19	26	1
	**Median**	-	32	45	72	90	98
	**Q1**	-	23	38	65	79.5	98
	**Q3**	-	-	60	78	92	98

PSI, Patient State Index; RASS, Richmond Agitation Sedation Scale

**Table 5. ROC Analysis: PSI with Respect to RASS 0 to -3:** 

**Regimen**		**Median PSI**	**IQR**		**PSI** **cut -off**	**Sensitivity**	**Specificity**	**PPV**	**NPV**	**AUC**	**95% CI AUC**		***P* value**
			**1^st^ Quartile**	**3^rd^** **Quartile**							**Lower bound**	**Upper bound**	
**Total**	**(n = 538)**	72	60	86	50.5	88.66	88.57	98.35	50.41	0.94	0.92	0.96	**<0.001**
	**(N = 35)**				51.4	100	100	-	100	1	0.83	1	**<0.001**
**Midazolam**	**(n = 249)**	73	59	88	57	78.31	100	100	12.9	0.92	0.87	0.97	**<0.001**
	**(N = 16)**				55	100	100	-	100	1	0.83	1	**<0.001**
**Midazolam + morphine**	**(n = 78)**	73.5	66	82	55.5	92.31	92.31	97.3	80	0.96	0.92	1	**<0.001**
	**(N = 6)**				57.4	100	100	-	100	1	0.69	1	**<0.001**
**Midazolam+fentanyl**	**(n = 38)**	67	56.5	76.25	All 38 observations were between 0 to -3 and no observations were noted in other group hence, analysis could not be performed.								
	**(N = 2)**												
**Morphine**	**(n = 118)**	66	56	80	57	88.98	91.18	97.22	70.45	0.95	0.91	0.98	**<0.001**
	**(N = 8)**				45.4	100	100	-	100	1	0.73	1	**<0.001**
**Fentanyl**	**(n = 55)**	78	65	90	41.5	96.36	100	100	50	0.98	0.94	1	**<0.001**
	**(N = 3)**				56	100	100	-	100	1	.48	1	**<0.001**

*: n represents total number of observations; N represents averaged values per patient

**: Given the small sample size in subgroup analyses, this result may reflect overfitting or statistical instability, rather than truly perfect predictive performance

PSI, Patient State Index; RASS, Richmond Agitation Sedation Scale; ROC, receiver operating characteristic; IQR, interquartile range; PPV, positive predictive value; NPV, negative predictive value; AUC, area under the curve; CI, confidence interval
